# Supplementary material for: Decoding gray matter, large-scale analysis of brain cell morphometry to inform microstructural modeling of diffusion MR signals
Source: Commun Biol. 2026 Jan 7;9:138. doi: 10.1038/s42003-025-09353-5 (PMC12858938; doi:10.1038/s42003-025-09353-5)
Supplement: Supplementary file 2 — Supplementary Information [file 42003_2025_9353_MOESM2_ESM.pdf]

Supplementary Material of: "Decoding Gray Matter: large-scale analysis of brain cell morphometry to inform microstructural modeling of diffusion MR signals"

Charlie Aird-Rossiter[1,2,*], Hui Zhang[3], Daniel C. Alexander[3], Derek K. Jones[1], and Marco Palombo[1,2,*]

[1]Cardiff University Brain Research Imaging Centre (CUBRIC), School of Psychology, Cardiff University, Cardiff, United Kingdom
[2]School of Computer Science and Informatics, Cardiff University, Cardiff, United Kingdom
[3]UCL Hawkes Institute and Department of Computer Science, University College London, London, United Kingdom
[*]Corresponding authors: Charlie Aird-Rossiter, aird-rossiterc@cardiff.ac.uk and Marco Palombo, palombom@cardiff.ac.uk

	Search criteria										Total number of cells
	Species	Development	Condition	Celltype	Structural Domain	Morphological Attributes	Number	Rejection rate (%)	total for species		11850
Accepted	Mouse	Adult, young, young adult	Control	Microglia	Dendrite, processes, soma, axon, no axon	Diameter, 3D, Angles	2696		5991		
Total	Mouse	Adult, young, young adult	Control	Microglia	n.a	n.a	27142	90.06705475			
Accepted	Mouse	Adult, young, young adult	Control	Astrocyte	Dendrite, processes, soma, axon, no axon	Diameter, 3D, Angles	197				
Total	Mouse	Adult, young, young adult	Control	Astrocyte	n.a	n.a	1607	87.74113255			
Accepted	Mouse	Adult, young, young adult	Control	Oligodendrocyte	Dendrite, processes, soma, axon, no axon	Diameter, 3D, Angles	53				
Total	Mouse	Adult, young, young adult	Control	Oligodendrocyte	n.a	n.a	122	56.55737705			
Accepted	Mouse	Adult, young, young adult	Control	Pyramidal	Dendrite, processes, soma, axon, no axon	Diameter, 3D, Angles	1660				
Total	Mouse	Adult, young, young adult	Control	Pyramidal	n.a	n.a	12865	87.09677419			
Accepted	Mouse	Adult, young, young adult	Control	Granule	Dendrite, processes, soma, axon, no axon	Diameter, 3D, Angles	772				
Total	Mouse	Adult, young, young adult	Control	Granule	n.a	n.a	2415	68.03312629			
Accepted	Mouse	Adult, young, young adult	Control	Purkinje	Dendrite, processes, soma, axon, no axon	Diameter, 3D, Angles	139				
Total	Mouse	Adult, young, young adult	Control	Purkinje	n.a	n.a	369	62.33062331			
Accepted	Mouse	Adult, young, young adult	Control	Basket	Dendrite, processes, soma, axon, no axon	Diameter, 3D, Angles	324				
Total	Mouse	Adult, young, young adult	Control	Basket	n.a	n.a	481	32.64033264			
Accepted	Mouse	Adult, young, young adult	Control	Gabaergic	Dendrite, processes, soma, axon, no axon	Diameter, 3D, Angles	122				
Total	Mouse	Adult, young, young adult	Control	Gabaergic	n.a	n.a	1462	91.65526676			
Accepted	Mouse	Adult, young, young adult	Control	Glutamaergic	Dendrite, processes, soma, axon, no axon	Diameter, 3D, Angles	28				
Total	Mouse	Adult, young, young adult	Control	Glutamaergic	n.a	n.a	33	15.15151515			
Accepted	Rat	Adult, young, young adult	Control	Microglia	Dendrite, porcesses, soma, axon, no axon	Diameter, 3D, Angles	1379		3010		
Total	Rat	Adult, young, young adult	Control	Microglia	n.a	n.a	4129	66.60208283			
Accepted	Rat	Adult, young, young adult	Control	Astrocyte	Dendrite, porcesses, soma, axon, no axon	Diameter, 3D, Angles	139				
Total	Rat	Adult, young, young adult	Control	Astrocyte	n.a	n.a	250	44.4			
Accepted	Rat	Adult, young, young adult	Control	Oligodendrocyte	Dendrite, porcesses, soma, axon, no axon	Diameter, 3D, Angles	5				
Total	Rat	Adult, young, young adult	Control	Oligodendrocyte	n.a	n.a	25	80			
Accepted	Rat	Adult, young, young adult	Control	Pyramidal	Dendrite, porcesses, soma, axon, no axon	Diameter, 3D, Angles	1150				
Total	Rat	Adult, young, young adult	Control	Pyramidal	n.a	n.a	2649	56.58739147			
Accepted	Rat	Adult, young, young adult	Control	Granule	Dendrite, porcesses, soma, axon, no axon	Diameter, 3D, Angles	112				
Total	Rat	Adult, young, young adult	Control	Granule	n.a	n.a	538	79.18215613			
Accepted	Rat	Adult, young, young adult	Control	Purkinje	Dendrite, porcesses, soma, axon, no axon	Diameter, 3D, Angles	1				
Total	Rat	Adult, young, young adult	Control	Purkinje	n.a	n.a	1	0			
Accepted	Rat	Adult, young, young adult	Control	Basket	Dendrite, porcesses, soma, axon, no axon	Diameter, 3D, Angles	90				
Total	Rat	Adult, young, young adult	Control	Basket	n.a	n.a	430	79.06976744			
Accepted	Rat	Adult, young, young adult	Control	Gabaergic	Dendrite, porcesses, soma, axon, no axon	Diameter, 3D, Angles	56				
Total	Rat	Adult, young, young adult	Control	Gabaergic	n.a	n.a	65	13.84615385			
Accepted	Rat	Adult, young, young adult	Control	Glutamatergic	Dendrite, porcesses, soma, axon, no axon	Diameter, 3D, Angles	78				
Total	Rat	Adult, young, young adult	Control	Glutamatergic	n.a	n.a	78	0			
Accepted	Monkey	Adult, young, young adult	Control	Microglia	Dendrite, porcesses, soma, axon, no axon	Diameter, 3D, Angles	60		525		
Total	Monkey	Adult, young, young adult	Control	Microglia	n.a	n.a	60	0			
Accepted	Monkey	Adult, young, young adult	Control	Astrocyte	Dendrite, porcesses, soma, axon, no axon	Diameter, 3D, Angles	0				
Total	Monkey	Adult, young, young adult	Control	Astrocyte	n.a	n.a	110	100			
Accepted	Monkey	Adult, young, young adult	Control	Oligodendrocyte	Dendrite, porcesses, soma, axon, no axon	Diameter, 3D, Angles	0				
Total	Monkey	Adult, young, young adult	Control	Oligodendrocyte	n.a	n.a	0	#DIV/0!			
Accepted	Monkey	Adult, young, young adult	Control	Pyramidal	Dendrite, porcesses, soma, axon, no axon	Diameter, 3D, Angles	461				
Total	Monkey	Adult, young, young adult	Control	Pyramidal	n.a	n.a	1736	73.44470046			
Accepted	Monkey	Adult, young, young adult	Control	Granule	Dendrite, porcesses, soma, axon, no axon	Diameter, 3D, Angles	0				
Total	Monkey	Adult, young, young adult	Control	Granule	n.a	n.a	0	#DIV/0!			
Accepted	Monkey	Adult, young, young adult	Control	Purkinje	Dendrite, porcesses, soma, axon, no axon	Diameter, 3D, Angles	0				
Total	Monkey	Adult, young, young adult	Control	Purkinje	n.a	n.a	0	#DIV/0!			
Accepted	Monkey	Adult, young, young adult	Control	Basket	Dendrite, porcesses, soma, axon, no axon	Diameter, 3D, Angles	0				
Total	Monkey	Adult, young, young adult	Control	Basket	n.a	n.a	9	100			
Accepted	Monkey	Adult, young, young adult	Control	Gabaergic	Dendrite, porcesses, soma, axon, no axon	Diameter, 3D, Angles	4				
Total	Monkey	Adult, young, young adult	Control	Gabaergic	n.a	n.a	4	0			
Accepted	Monkey	Adult, young, young adult	Control	Glutamatergic	Dendrite, porcesses, soma, axon, no axon	Diameter, 3D, Angles	0				
Total	Monkey	Adult, young, young adult	Control	Glutamatergic	n.a	n.a	0	#DIV/0!			

Figure 1: **Table displaying the search criteria and resulting number of accepted reconstructions and corresponding rejection rate**

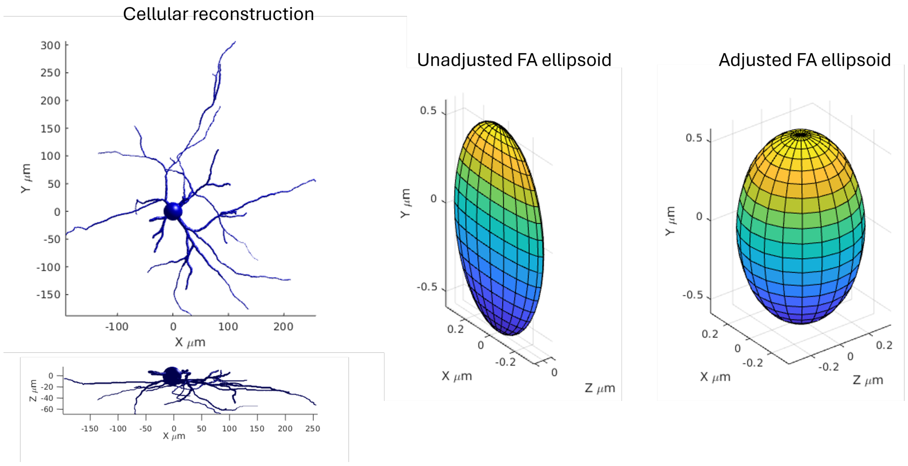

Figure 2: **Figure demonstrating the limit depth of cellular reconstructions as a result of imaging method (some reconstructions appeared even more ‘flattened’), and the resulting FA tensors for unadjusted eigenvalues and adjusted eigenvalues. The corresponding FA for unadjusted eigenvalues = 0.67, and for adjusted eigenvalues = 0.30, so reduced dimensional representation results in increased FA.**

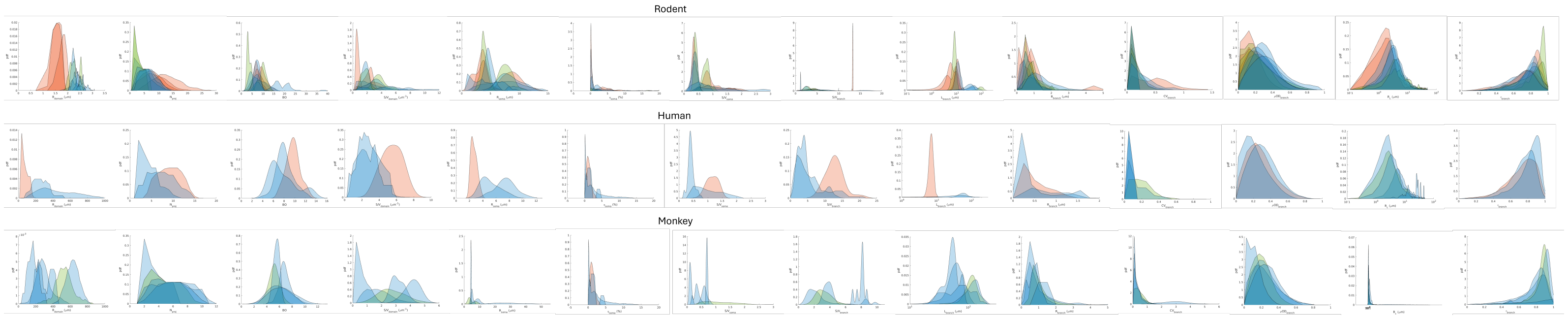

Figure 3: **Structural feature distribution for all species**

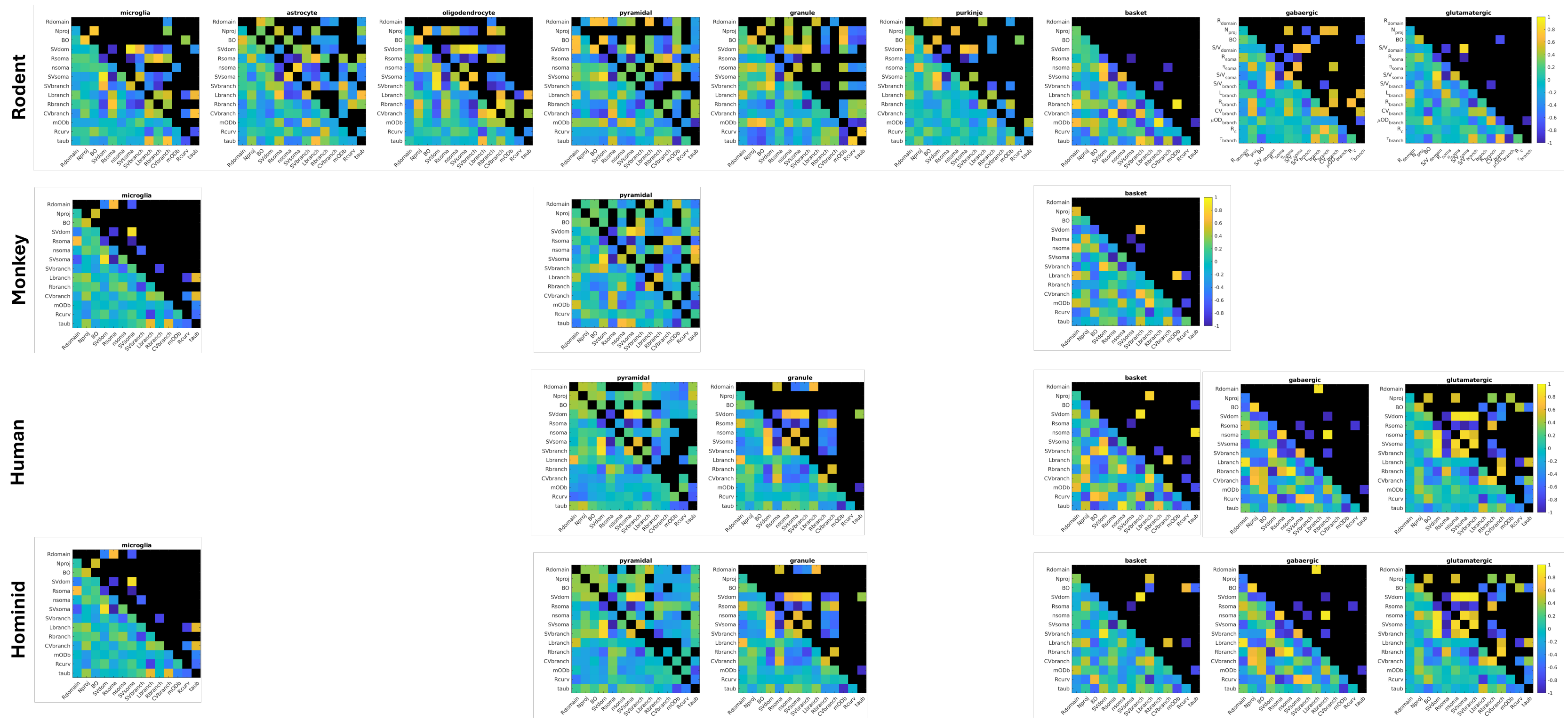

Figure 4: **Spearman's rank correlation between structural features for all species and cell types** Cell colour indicates the strength of the correlation and black cells indicate correlations that were found not to be significant (p-value adjusted according to Bonferroni correction)

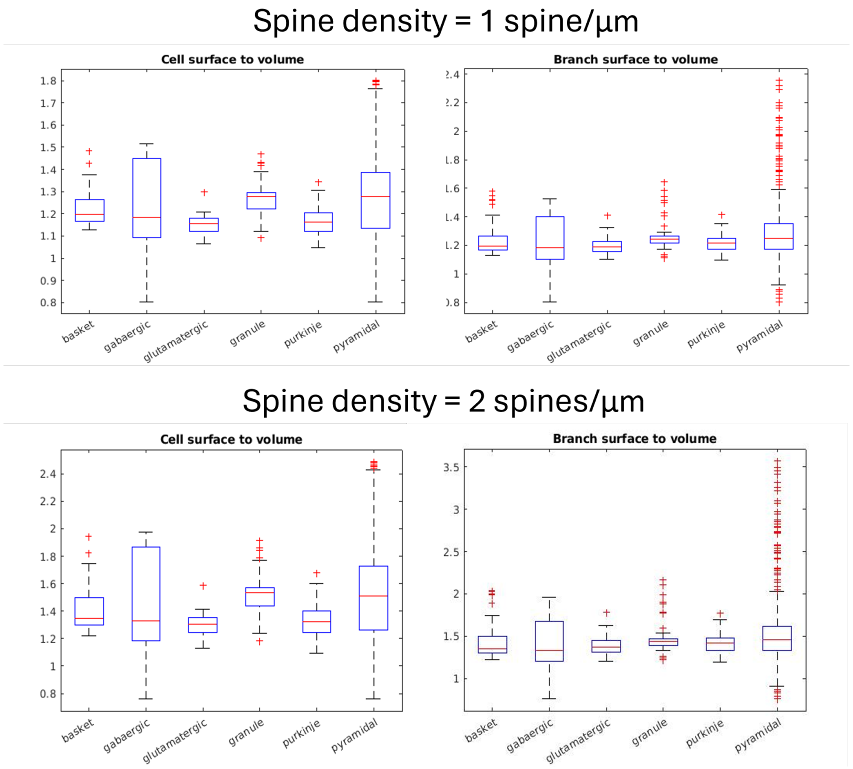

Figure 5: **Estimated impact of dendritic spines on surface-to-volume ratios** for rodent cell types, for densities of one and two $spines/\mu m$.

Spine modeling

To estimate the impact of dendritic spines on the surface-to-volume ratio, and the resulting effects on molecular residence and exchange times, the total dendritic length for each individual cell was calculated. Based on the measured dendritic length and the prescribed spine density (1 and 2 $spine\mu m^{-1}$), the number of spines, n, per cell was determined. Given the surface area, S_{spine}, and volume, V_{spine}, for a single dendritic spine for each species (as reported in the literature, Karbowski and Urban 2023), we added the corresponding total spine surface (nS_{spine}) and volume (nV_{spine}) to the cellular surface and volume, respectively. Finally, we recalculated the surface-to-volume ratios, including the contribution of dendritic spines, and the corresponding change in surface-to-volume ratio (Fig.5).

References

Karbowski, Jan and Paulina Urban (2023). "Information encoded in volumes and areas of dendritic spines is nearly maximal across mammalian brains". In: *Scientific Reports* 13.1, p. 22207.

Neuromorpho.org Archives

Here we cite the articles the reconstructions were obtained from, as referenced in Neuromorpho.org.

References

Aalling, Nadia et al. (July 2017). "Erythropoietin prevents the effect of chronic restraint stress on the number of hippocampal CA3c dendritic terminals—relation to expression of genes involved in synaptic plasticity, angiogenesis, inflammation, and oxidative stress in male rats". In: *Journal of Neuroscience Research* 96.1, pp. 103–116. ISSN: 1097-4547. DOI: 10.1002/jnr.24107. URL: http://dx.doi.org/10.1002/jnr.24107.

Abdolhoseini, Mahmoud et al. (June 2019). "Segmentation, Tracing, and Quantification of Microglial Cells from 3D Image Stacks". In: *Scientific Reports* 9.1. ISSN: 2045-2322. DOI: 10.1038/s41598-019-44917-6. URL: http://dx.doi.org/10.1038/s41598-019-44917-6.

Acker, Corey D. and Srdjan D. Antic (Mar. 2009). "Quantitative Assessment of the Distributions of Membrane Conductances Involved in Action Potential Backpropagation Along Basal Dendrites". In: *Journal of Neurophysiology* 101.3, pp. 1524–1541. ISSN: 1522-1598. DOI: 10.1152/jn.00651.2007. URL: http://dx.doi.org/10.1152/jn.00651.2007.

Allen, Barrett D. et al. (May 2020). "Mitigation of helium irradiation-induced brain injury by microglia depletion". In: *Journal of Neuroinflammation* 17.1. ISSN: 1742-2094. DOI: 10.1186/s12974-020-01790-9. URL: http://dx.doi.org/10.1186/s12974-020-01790-9.

Althammer, Ferdinand et al. (July 2020). "Three-dimensional morphometric analysis reveals time-dependent structural changes in microglia and astrocytes in the central amygdala and hypothalamic paraventricular nucleus of heart failure rats". In: *Journal of Neuroinflammation* 17.1. ISSN: 1742-2094. DOI: 10.1186/s12974-020-01892-4. URL: http://dx.doi.org/10.1186/s12974-020-01892-4.

Anderson, Rachel M. et al. (May 2016). "Prolonged corticosterone exposure induces dendritic spine remodeling and attrition in the rat medial prefrontal cortex". In: *Journal of Comparative Neurology* 524.18, pp. 3729–3746. ISSN: 1096-9861. DOI: 10.1002/cne.24027. URL: http://dx.doi.org/10.1002/cne.24027.

Anstötz, Max and Gianmaria Maccaferri (Jan. 2020). “A Toolbox of Criteria for Distinguishing Cajal–Retzius Cells from Other Neuronal Types in the Postnatal Mouse Hippocampus”. In: *eneuro* 7.1, ENEURO.0516–19.2019. ISSN: 2373-2822. DOI: 10.1523/eneuro.0516-19.2019. URL: http://dx.doi.org/10.1523/ENEURO.0516-19.2019.

Anwar, Haroon et al. (July 2014). “Dendritic diameters affect the spatial variability of intracellular calcium dynamics in computer models”. In: *Frontiers in Cellular Neuroscience* 8. ISSN: 1662-5102. DOI: 10.3389/fncel.2014.00168. URL: http://dx.doi.org/10.3389/fncel.2014.00168.

Arruda-Carvalho, Maithe et al. (Sept. 2014). “Conditional Deletion of -CaMKII Impairs Integration of Adult-Generated Granule Cells into Dentate Gyrus Circuits and Hippocampus-Dependent Learning”. In: *The Journal of Neuroscience* 34.36, pp. 11919–11928. ISSN: 1529-2401. DOI: 10.1523/jneurosci.0652-14.2014. URL: http://dx.doi.org/10.1523/JNEUROSCI.0652-14.2014.

Arvin, Matthew C. et al. (Mar. 2019). “Chronic Nicotine Exposure Alters the Neurophysiology of Habenulo-Interpeduncular Circuitry”. In: *The Journal of Neuroscience* 39.22, pp. 4268–4281. ISSN: 1529-2401. DOI: 10.1523/jneurosci.2816-18.2019. URL: http://dx.doi.org/10.1523/JNEUROSCI.2816-18.2019.

Assous, Maxime et al. (May 2018). “Identification and Characterization of a Novel Spontaneously Active Bursty GABAergic Interneuron in the Mouse Striatum”. In: *The Journal of Neuroscience* 38.25, pp. 5688–5699. ISSN: 1529-2401. DOI: 10.1523/jneurosci.3354-17.2018. URL: http://dx.doi.org/10.1523/JNEUROSCI.3354-17.2018.

Ativie, Frank et al. (Aug. 2018). “Cannabinoid 1 Receptor Signaling on Hippocampal GABAergic Neurons Influences Microglial Activity”. In: *Frontiers in Molecular Neuroscience* 11. ISSN: 1662-5099. DOI: 10.3389/fnmol.2018.00295. URL: http://dx.doi.org/10.3389/fnmol.2018.00295.

Bailey, Craig D.C. et al. (Feb. 2014). “Chrna5 genotype determines the long-lasting effects of developmental in vivo nicotine exposure on prefrontal attention circuitry”. In: *Neuropharmacology* 77, pp. 145–155. ISSN: 0028-3908. DOI: 10.1016/j.neuropharm.2013.09.003. URL: http://dx.doi.org/10.1016/j.neuropharm.2013.09.003.

Bao, Hechen et al. (Nov. 2017). “Long-Range GABAergic Inputs Regulate Neural Stem Cell Quiescence and Control Adult Hippocampal Neurogenesis”. In: *Cell Stem Cell* 21.5, 604–617.e5. ISSN: 1934-5909. DOI: 10.1016/j.stem.2017.10.003. URL: http://dx.doi.org/10.1016/j.stem.2017.10.003.

Bathellier, Brice, Troy W. Margrie, and Matthew E. Larkum (Oct. 2009). “Properties of Piriform Cortex Pyramidal Cell Dendrites: Implications for Olfactory Circuit Design”. In: *The Journal of Neuroscience* 29.40, pp. 12641–12652. ISSN: 1529-2401. DOI: 10.1523/jneurosci.1124-09.2009. URL: http://dx.doi.org/10.1523/JNEUROSCI.1124-09.2009.

Benavides-Piccione, Ruth, Jon I. Arellano, and Javier DeFelipe (Feb. 2005). “Catecholaminergic Innervation of Pyramidal Neurons in the Human Temporal Cortex”. In: *Cerebral Cortex* 15.10, pp. 1584–1591. ISSN: 1047-3211.

DOI: 10.1093/cercor/bhi036. URL: http://dx.doi.org/10.1093/cercor/bhi036.

Bezchlibnyk, Yarema B. et al. (Mar. 2017). "High frequency stimulation of the infralimbic cortex induces morphological changes in rat hippocampal neurons". In: *Brain Stimulation* 10.2, pp. 315–323. ISSN: 1935-861X. DOI: 10.1016/j.brs.2016.11.013. URL: http://dx.doi.org/10.1016/j.brs.2016.11.013.

Bienvenu, Thomas C.M. et al. (Feb. 2015). "Large Intercalated Neurons of Amygdala Relay Noxious Sensory Information". In: *The Journal of Neuroscience* 35.5, pp. 2044–2057. ISSN: 1529-2401. DOI: 10.1523/jneurosci.1323-14.2015. URL: http://dx.doi.org/10.1523/JNEUROSCI.1323-14.2015.

Blackman, Arne V. et al. (July 2014). "A comparison of manual neuronal reconstruction from biocytin histology or 2-photon imaging: morphometry and computer modeling". In: *Frontiers in Neuroanatomy* 8. ISSN: 1662-5129. DOI: 10.3389/fnana.2014.00065. URL: http://dx.doi.org/10.3389/fnana.2014.00065.

Bland, Katherine M. et al. (Mar. 2021). "FMRP regulates the subcellular distribution of cortical dendritic spine density in a non-cell-autonomous manner". In: *Neurobiology of Disease* 150, p. 105253. ISSN: 0969-9961. DOI: 10.1016/j.nbd.2021.105253. URL: http://dx.doi.org/10.1016/j.nbd.2021.105253.

Bloss, Erik B. et al. (Mar. 2016). "Structured Dendritic Inhibition Supports Branch-Selective Integration in CA1 Pyramidal Cells". In: *Neuron* 89.5, pp. 1016–1030. ISSN: 0896-6273. DOI: 10.1016/j.neuron.2016.01.029. URL: http://dx.doi.org/10.1016/j.neuron.2016.01.029.

Blume, Shannon R. et al. (Sept. 2017). "Sex- and Estrus-Dependent Differences in Rat Basolateral Amygdala". In: *The Journal of Neuroscience* 37.44, pp. 10567–10586. ISSN: 1529-2401. DOI: 10.1523/jneurosci.0758-17.2017. URL: http://dx.doi.org/10.1523/JNEUROSCI.0758-17.2017.

Bories, Cyril et al. (Jan. 2013). "Differential Balance of Prefrontal Synaptic Activity in Successful versus Unsuccessful Cognitive Aging". In: *The Journal of Neuroscience* 33.4, pp. 1344–1356. ISSN: 1529-2401. DOI: 10.1523/jneurosci.3258-12.2013. URL: http://dx.doi.org/10.1523/JNEUROSCI.3258-12.2013.

Boudewijns, Zimbo S. R. M. et al. (2013). "Layer-specific high-frequency action potential spiking in the prefrontal cortex of awake rats". In: *Frontiers in Cellular Neuroscience* 7. ISSN: 1662-5102. DOI: 10.3389/fncel.2013.00099. URL: http://dx.doi.org/10.3389/fncel.2013.00099.

Briggs, Farran et al. (Apr. 2016). "Morphological Substrates for Parallel Streams of Corticogeniculate Feedback Originating in Both V1 and V2 of the Macaque Monkey". In: *Neuron* 90.2, pp. 388–399. ISSN: 0896-6273. DOI: 10.1016/j.neuron.2016.02.038. URL: http://dx.doi.org/10.1016/j.neuron.2016.02.038.

Brown, Kerry M. et al. (2005). "A Cross-Platform Freeware Tool for Digital Reconstruction of Neuronal Arborizations From Image Stacks". In: *Neu-*

roinformatics 3.4, pp. 343–360. ISSN: 1539-2791. DOI: 10.1385/ni:3:4:343. URL: http://dx.doi.org/10.1385/NI:3:4:343.

Buchin, Anatoly et al. (Dec. 2022). "Multi-modal characterization and simulation of human epileptic circuitry". In: *Cell Reports* 41.13, p. 111873. ISSN: 2211-1247. DOI: 10.1016/j.celrep.2022.111873. URL: http://dx.doi.org/10.1016/j.celrep.2022.111873.

Cadwell, Cathryn R et al. (Mar. 2020). "Cell type composition and circuit organization of clonally related excitatory neurons in the juvenile mouse neocortex". In: *eLife* 9. ISSN: 2050-084X. DOI: 10.7554/elife.52951. URL: http://dx.doi.org/10.7554/elife.52951.

Carim-Todd, Laura et al. (Jan. 2009). "Endogenous Truncated TrkB.T1 Receptor Regulates Neuronal Complexity and TrkB Kinase Receptor FunctionIn Vivo". In: *The Journal of Neuroscience* 29.3, pp. 678–685. ISSN: 1529-2401. DOI: 10.1523/jneurosci.5060-08.2009. URL: http://dx.doi.org/10.1523/JNEUROSCI.5060-08.2009.

Çetereisi, Demirhan et al. (Dec. 2018). "Gpr158 deficiency impacts hippocampal CA1 neuronal excitability, dendritic architecture, and affects spatial learning". In: DOI: 10.1101/506295. URL: http://dx.doi.org/10.1101/506295.

Chailangkarn, Thanathom et al. (Aug. 2016). "A human neurodevelopmental model for Williams syndrome". In: *Nature* 536.7616, pp. 338–343. ISSN: 1476-4687. DOI: 10.1038/nature19067. URL: http://dx.doi.org/10.1038/nature19067.

Chen, Xiao Ru et al. (May 2013). "Mature Purkinje Cells Require the Retinoic Acid-Related Orphan Receptor- (ROR) to Maintain Climbing Fiber Mono-Innervation and Other Adult Characteristics". In: *The Journal of Neuroscience* 33.22, pp. 9546–9562. ISSN: 1529-2401. DOI: 10.1523/jneurosci.2977-12.2013. URL: http://dx.doi.org/10.1523/JNEUROSCI.2977-12.2013.

Chu, Hong-Yuan et al. (Sept. 2017). "Loss of Hyperdirect Pathway Cortico-Subthalamic Inputs Following Degeneration of Midbrain Dopamine Neurons". In: *Neuron* 95.6, 1306–1318.e5. ISSN: 0896-6273. DOI: 10.1016/j.neuron.2017.08.038. URL: http://dx.doi.org/10.1016/j.neuron.2017.08.038.

Colombo, Gloria et al. (Dec. 2021). "Microglial MorphOMICs unravel region- and sex-dependent morphological phenotypes from postnatal development to degeneration". In: DOI: 10.1101/2021.11.30.470610. URL: http://dx.doi.org/10.1101/2021.11.30.470610.

Courcelles, Erik Justin et al. (Apr. 2024). "Association cortical areas in the mouse contain a large population of fast-spiking GABAergic neurons that do not express parvalbumin". In: *European Journal of Neuroscience* 59.12, pp. 3236–3255. ISSN: 1460-9568. DOI: 10.1111/ejn.16341. URL: http://dx.doi.org/10.1111/ejn.16341.

Dieni, Cristina V. et al. (Dec. 2013). "Distinct Determinants of Sparse Activation during Granule Cell Maturation". In: *The Journal of Neuroscience* 33.49, pp. 19131–19142. ISSN: 1529-2401. DOI: 10.1523/jneurosci.2289-13.2013. URL: http://dx.doi.org/10.1523/JNEUROSCI.2289-13.2013.

Diniz, Daniel Guerreiro et al. (Oct. 2016). "Age, environment, object recognition and morphological diversity of GFAP-immunolabeled astrocytes". In: *Behavioral and Brain Functions* 12.1. ISSN: 1744-9081. DOI: 10.1186/s12993-016-0111-2. URL: http://dx.doi.org/10.1186/s12993-016-0111-2.

Duan, H et al. (Oct. 2002). "Quantitative analysis of the dendritic morphology of corticocortical projection neurons in the macaque monkey association cortex". In: *Neuroscience* 114.2, pp. 349–359. ISSN: 0306-4522. DOI: 10.1016/s0306-4522(02)00305-6. URL: http://dx.doi.org/10.1016/S0306-4522(02)00305-6.

Elston, Guy N. et al. (2011). "Spinogenesis and Pruning in the Anterior Ventral Inferotemporal Cortex of the Macaque Monkey: An Intracellular Injection Study of Layer III Pyramidal Cells". In: *Frontiers in Neuroanatomy* 5. ISSN: 1662-5129. DOI: 10.3389/fnana.2011.00042. URL: http://dx.doi.org/10.3389/fnana.2011.00042.

Errico, F et al. (July 2014). "Free D-aspartate regulates neuronal dendritic morphology, synaptic plasticity, gray matter volume and brain activity in mammals". In: *Translational Psychiatry* 4.7, e417–e417. ISSN: 2158-3188. DOI: 10.1038/tp.2014.59. URL: http://dx.doi.org/10.1038/tp.2014.59.

Eyal, Guy et al. (Oct. 2016). "Unique membrane properties and enhanced signal processing in human neocortical neurons". In: *eLife* 5. ISSN: 2050-084X. DOI: 10.7554/elife.16553. URL: http://dx.doi.org/10.7554/eLife.16553.

Francavilla, Ruggiero et al. (Nov. 2018). "Connectivity and network state-dependent recruitment of long-range VIP-GABAergic neurons in the mouse hippocampus". In: *Nature Communications* 9.1. ISSN: 2041-1723. DOI: 10.1038/s41467-018-07162-5. URL: http://dx.doi.org/10.1038/s41467-018-07162-5.

Gliko, Olga et al. (July 2024). "High-throughput analysis of dendrite and axonal arbors reveals transcriptomic correlates of neuroanatomy". In: *Nature Communications* 15.1. ISSN: 2041-1723. DOI: 10.1038/s41467-024-50728-9. URL: http://dx.doi.org/10.1038/s41467-024-50728-9.

Gong, Hui et al. (July 2016). "High-throughput dual-colour precision imaging for brain-wide connectome with cytoarchitectonic landmarks at the cellular level". In: *Nature Communications* 7.1. ISSN: 2041-1723. DOI: 10.1038/ncomms12142. URL: http://dx.doi.org/10.1038/ncomms12142.

Gonzalez-Burgos, G. (Mar. 2004). "Synaptic Efficacy during Repetitive Activation of Excitatory Inputs in Primate Dorsolateral Prefrontal Cortex". In: *Cerebral Cortex* 14.5, pp. 530–542. ISSN: 1460-2199. DOI: 10.1093/cercor/bhh015. URL: http://dx.doi.org/10.1093/cercor/bhh015.

Groen, Martine R. et al. (July 2014). "Development of dendritic tonic GABAergic inhibition regulates excitability and plasticity in CA1 pyramidal neurons". In: *Journal of Neurophysiology* 112.2, pp. 287–299. ISSN: 1522-1598. DOI: 10.1152/jn.00066.2014. URL: http://dx.doi.org/10.1152/jn.00066.2014.

Halnes, Geir et al. (Sept. 2011). "A Multi-Compartment Model for Interneurons in the Dorsal Lateral Geniculate Nucleus". In: *PLoS Computational Biology*

7.9. Ed. by Olaf Sporns, e1002160. ISSN: 1553-7358. DOI: 10.1371/journal.pcbi.1002160. URL: http://dx.doi.org/10.1371/journal.pcbi.1002160.

Her, Lu-Shiun et al. (2017). "miR-196a Enhances Neuronal Morphology through Suppressing RANBP10 to Provide Neuroprotection in Huntington's Disease". In: *Theranostics* 7.9, pp. 2452–2462. ISSN: 1838-7640. DOI: 10.7150/thno.18813. URL: http://dx.doi.org/10.7150/thno.18813.

Hernandez, Michael X. et al. (Sept. 2017). "Prevention of C5aR1 signaling delays microglial inflammatory polarization, favors clearance pathways and suppresses cognitive loss". In: *Molecular Neurodegeneration* 12.1. ISSN: 1750-1326. DOI: 10.1186/s13024-017-0210-z. URL: http://dx.doi.org/10.1186/s13024-017-0210-z.

Hosp, Jonas A. et al. (Oct. 2013). "Morpho-physiological criteria divide dentate gyrus interneurons into classes". In: *Hippocampus* 24.2, pp. 189–203. ISSN: 1098-1063. DOI: 10.1002/hipo.22214. URL: http://dx.doi.org/10.1002/hipo.22214.

Hsu, Tsan-Ting et al. (June 2015). "Differential Recruitment of Dentate Gyrus Interneuron Types by Commissural Versus Perforant Pathways". In: *Cerebral Cortex* 26.6, pp. 2715–2727. ISSN: 1460-2199. DOI: 10.1093/cercor/bhv127. URL: http://dx.doi.org/10.1093/cercor/bhv127.

Iascone, Daniel Maxim et al. (May 2020). "Whole-Neuron Synaptic Mapping Reveals Spatially Precise Excitatory/Inhibitory Balance Limiting Dendritic and Somatic Spiking". In: *Neuron* 106.4, 566–578.e8. ISSN: 0896-6273. DOI: 10.1016/j.neuron.2020.02.015. URL: http://dx.doi.org/10.1016/j.neuron.2020.02.015.

Jaarsma, Dick et al. (Sept. 2018). "The basal interstitial nucleus (BIN) of the cerebellum provides diffuse ascending inhibitory input to the floccular granule cell layer". In: *Journal of Comparative Neurology* 526.14, pp. 2231–2256. ISSN: 1096-9861. DOI: 10.1002/cne.24479. URL: http://dx.doi.org/10.1002/cne.24479.

Jacobs, Bob, Madeleine E. Garcia, et al. (Nov. 2017). "Comparative morphology of gigantopyramidal neurons in primary motor cortex across mammals". In: *Journal of Comparative Neurology* 526.3, pp. 496–536. ISSN: 1096-9861. DOI: 10.1002/cne.24349. URL: http://dx.doi.org/10.1002/cne.24349.

Jacobs, Bob, Nicholas L. Johnson, et al. (Apr. 2014). "Comparative neuronal morphology of the cerebellar cortex in afrotherians, carnivores, cetartiodactyls, and primates". In: *Frontiers in Neuroanatomy* 8. ISSN: 1662-5129. DOI: 10.3389/fnana.2014.00024. URL: http://dx.doi.org/10.3389/fnana.2014.00024.

Jacot-Descombes, Sarah et al. (Nov. 2020). "Altered synaptic ultrastructure in the prefrontal cortex of Shank3-deficient rats". In: *Molecular Autism* 11.1. ISSN: 2040-2392. DOI: 10.1186/s13229-020-00393-8. URL: http://dx.doi.org/10.1186/s13229-020-00393-8.

Jayabal, Sriram, Lovisa Ljungberg, and Alanna J. Watt (Oct. 2016). "Transient cerebellar alterations during development prior to obvious motor phenotype in a mouse model of spinocerebellar ataxia type 6". In: *The Journal of Phys-*

iology 595.3, pp. 949–966. ISSN: 1469-7793. DOI: 10.1113/jp273184. URL: http://dx.doi.org/10.1113/JP273184.

Jiang, Xiaolong et al. (Nov. 2015). "Principles of connectivity among morphologically defined cell types in adult neocortex". In: *Science* 350.6264. ISSN: 1095-9203. DOI: 10.1126/science.aac9462. URL: http://dx.doi.org/10.1126/science.aac9462.

Jongbloets, Bart C. et al. (Mar. 2017). "Stage-specific functions of Semaphorin7A during adult hippocampal neurogenesis rely on distinct receptors". In: *Nature Communications* 8.1. ISSN: 2041-1723. DOI: 10.1038/ncomms14666. URL: http://dx.doi.org/10.1038/ncomms14666.

Karlsson, Tobias E. et al. (Feb. 2016). "NgR1: A Tunable Sensor Regulating Memory Formation, Synaptic, and Dendritic Plasticity". In: *Cerebral Cortex* 26.4, pp. 1804–1817. ISSN: 1460-2199. DOI: 10.1093/cercor/bhw007. URL: http://dx.doi.org/10.1093/cercor/bhw007.

Ke, Meng-Tsen et al. (Mar. 2016). "Super-Resolution Mapping of Neuronal Circuitry With an Index-Optimized Clearing Agent". In: *Cell Reports* 14.11, pp. 2718–2732. ISSN: 2211-1247. DOI: 10.1016/j.celrep.2016.02.057. URL: http://dx.doi.org/10.1016/j.celrep.2016.02.057.

Keyvani, Kathy et al. (Apr. 2018). "Higher levels of kallikrein-8 in female brain may increase the risk for Alzheimer's disease". In: *Brain Pathology* 28.6, pp. 947–964. ISSN: 1750-3639. DOI: 10.1111/bpa.12599. URL: http://dx.doi.org/10.1111/bpa.12599.

Kiyota, Tomomi et al. (May 2018). "URMC-099 facilitates amyloid- clearance in a murine model of Alzheimer's disease". In: *Journal of Neuroinflammation* 15.1. ISSN: 1742-2094. DOI: 10.1186/s12974-018-1172-y. URL: http://dx.doi.org/10.1186/s12974-018-1172-y.

Ko, Meng-Ching et al. (May 2014). "Long-term consequences of neonatal fluoxetine exposure in adult rats". In: *Developmental Neurobiology* 74.10, pp. 1038–1051. ISSN: 1932-846X. DOI: 10.1002/dneu.22185. URL: http://dx.doi.org/10.1002/dneu.22185.

Koch, Christof and Allan Jones (Nov. 2016). "Big Science, Team Science, and Open Science for Neuroscience". In: *Neuron* 92.3, pp. 612–616. ISSN: 0896-6273. DOI: 10.1016/j.neuron.2016.10.019. URL: http://dx.doi.org/10.1016/j.neuron.2016.10.019.

Koelbl, Christian et al. (Sept. 2013). "A Barrel-Related Interneuron in Layer 4 of Rat Somatosensory Cortex with a High Intrabarrel Connectivity". In: *Cerebral Cortex* 25.3, pp. 713–725. ISSN: 1047-3211. DOI: 10.1093/cercor/bht263. URL: http://dx.doi.org/10.1093/cercor/bht263.

Komulainen, Emilia et al. (Sept. 2014). "JNK1 controls dendritic field size in L2/3 and L5 of the motor cortex, constrains soma size, and influences fine motor coordination". In: *Frontiers in Cellular Neuroscience* 8. ISSN: 1662-5102. DOI: 10.3389/fncel.2014.00272. URL: http://dx.doi.org/10.3389/fncel.2014.00272.

Kovács, Adrienn and Balázs Pál (Feb. 2017). "Astrocyte-Dependent Slow Inward Currents (SICs) Participate in Neuromodulatory Mechanisms in the Pedunculopontine Nucleus (PPN)". In: *Frontiers in Cellular Neuroscience*

11. ISSN: 1662-5102. DOI: 10.3389/fncel.2017.00016. URL: http://dx.doi.org/10.3389/fncel.2017.00016.

Kubota, Yoshiyuki et al. (July 2015). "Functional effects of distinct innervation styles of pyramidal cells by fast spiking cortical interneurons". In: *eLife* 4. ISSN: 2050-084X. DOI: 10.7554/elife.07919. URL: http://dx.doi.org/10.7554/eLife.07919.

Kula, Joanna et al. (July 2017). "Diverse action of repeated corticosterone treatment on synaptic transmission, neuronal plasticity, and morphology in superficial and deep layers of the rat motor cortex". In: *Pflügers Archiv - European Journal of Physiology* 469.11, pp. 1519–1532. ISSN: 1432-2013. DOI: 10.1007/s00424-017-2036-5. URL: http://dx.doi.org/10.1007/s00424-017-2036-5.

Kyrargyri, Vasiliki et al. (Sept. 2019). "P2Y13 receptors regulate microglial morphology, surveillance, and resting levels of interleukin 1 release". In: *Glia* 68.2, pp. 328–344. ISSN: 1098-1136. DOI: 10.1002/glia.23719. URL: http://dx.doi.org/10.1002/glia.23719.

Lalanne, Txomin et al. (Dec. 2015). "Synapse-specific expression of calcium-permeable AMPA receptors in neocortical layer 5". In: *The Journal of Physiology* 594.4, pp. 837–861. ISSN: 1469-7793. DOI: 10.1113/jp271394. URL: http://dx.doi.org/10.1113/JP271394.

Lee, Sang-Hun et al. (June 2014). "Parvalbumin-Positive Basket Cells Differentiate among Hippocampal Pyramidal Cells". In: *Neuron* 82.5, pp. 1129–1144. ISSN: 0896-6273. DOI: 10.1016/j.neuron.2014.03.034. URL: http://dx.doi.org/10.1016/j.neuron.2014.03.034.

Li, Yiding et al. (Mar. 2017). "A distinct entorhinal cortex to hippocampal CA1 direct circuit for olfactory associative learning". In: *Nature Neuroscience* 20.4, pp. 559–570. ISSN: 1546-1726. DOI: 10.1038/nn.4517. URL: http://dx.doi.org/10.1038/nn.4517.

Ligneul, Clémence et al. (May 2019). "Diffusion-weighted magnetic resonance spectroscopy enables cell-specific monitoring of astrocyte reactivity in vivo". In: *NeuroImage* 191, pp. 457–469. ISSN: 1053-8119. DOI: 10.1016/j.neuroimage.2019.02.046. URL: http://dx.doi.org/10.1016/j.neuroimage.2019.02.046.

Linaro, Daniele, Matthew J. Levy, and David L. Hunt (Apr. 2022). "Cell type-specific mechanisms of information transfer in data-driven biophysical models of hippocampal CA3 principal neurons". In: *PLOS Computational Biology* 18.4. Ed. by Michele Migliore, e1010071. ISSN: 1553-7358. DOI: 10.1371/journal.pcbi.1010071. URL: http://dx.doi.org/10.1371/journal.pcbi.1010071.

López-Doménech, Guillermo et al. (Oct. 2016). "Loss of Dendritic Complexity Precedes Neurodegeneration in a Mouse Model with Disrupted Mitochondrial Distribution in Mature Dendrites". In: *Cell Reports* 17.2, pp. 317–327. ISSN: 2211-1247. DOI: 10.1016/j.celrep.2016.09.004. URL: http://dx.doi.org/10.1016/j.celrep.2016.09.004.

Louth, Emma L. et al. (Jan. 2018). "Developmental ethanol exposure alters the morphology of mouse prefrontal neurons in a layer-specific manner".

In: *Brain Research* 1678, pp. 94–105. ISSN: 0006-8993. DOI: 10.1016/j.brainres.2017.10.005. URL: http://dx.doi.org/10.1016/j.brainres.2017.10.005.

Mańko, Mirosława, Raffaella Geracitano, and Marco Capogna (Apr. 2011). "Functional connectivity of the main intercalated nucleus of the mouse amygdala". In: *The Journal of Physiology* 589.8, pp. 1911–1925. ISSN: 1469-7793. DOI: 10.1113/jphysiol.2010.201475. URL: http://dx.doi.org/10.1113/jphysiol.2010.201475.

Markwardt, Sean J et al. (Oct. 2011). "Ivy/neurogliaform interneurons coordinate activity in the neurogenic niche". In: *Nature Neuroscience* 14.11, pp. 1407–1409. ISSN: 1546-1726. DOI: 10.1038/nn.2935. URL: http://dx.doi.org/10.1038/nn.2935.

Martone, Maryann E. et al. (2003). "The Cell-Centered Database: A Database for Multiscale Structural and Protein Localization Data from Light and Electron Microscopy". In: *Neuroinformatics* 1.4, pp. 379–396. ISSN: 1539-2791. DOI: 10.1385/ni:1:4:379. URL: http://dx.doi.org/10.1385/NI:1:4:379.

Marx, Manuel et al. (Dec. 2015). "Neocortical Layer 6B as a Remnant of the Subplate - A Morphological Comparison". In: *Cerebral Cortex*, bhv279. ISSN: 1460-2199. DOI: 10.1093/cercor/bhv279. URL: http://dx.doi.org/10.1093/cercor/bhv279.

Matta, Jose A et al. (July 2013). "Developmental origin dictates interneuron AMPA and NMDA receptor subunit composition and plasticity". In: *Nature Neuroscience* 16.8, pp. 1032–1041. ISSN: 1546-1726. DOI: 10.1038/nn.3459. URL: http://dx.doi.org/10.1038/nn.3459.

Medalla, Maria et al. (Apr. 2020). "Treatment with Mesenchymal-Derived Extracellular Vesicles Reduces Injury-Related Pathology in Pyramidal Neurons of Monkey Perilesional Ventral Premotor Cortex". In: *The Journal of Neuroscience* 40.17, pp. 3385–3407. ISSN: 1529-2401. DOI: 10.1523/jneurosci.2226-19.2020. URL: http://dx.doi.org/10.1523/JNEUROSCI.2226-19.2020.

Megías, M et al. (Feb. 2001). "Total number and distribution of inhibitory and excitatory synapses on hippocampal CA1 pyramidal cells". In: *Neuroscience* 102.3, pp. 527–540. ISSN: 0306-4522. DOI: 10.1016/s0306-4522(00)00496-6. URL: http://dx.doi.org/10.1016/S0306-4522(00)00496-6.

Meszéna, Domokos et al. (Jan. 2023). "Seeing beyond the spikes: reconstructing the complete spatiotemporal membrane potential distribution from paired intra- and extracellular recordings". In: *The Journal of Physiology* 601.15, pp. 3351–3376. ISSN: 1469-7793. DOI: 10.1113/jp283550. URL: http://dx.doi.org/10.1113/JP283550.

Meyer, Hanno S. et al. (June 2010). "Cell Type–Specific Thalamic Innervation in a Column of Rat Vibrissal Cortex". In: *Cerebral Cortex* 20.10, pp. 2287–2303. ISSN: 1047-3211. DOI: 10.1093/cercor/bhq069. URL: http://dx.doi.org/10.1093/cercor/bhq069.

Molnár, Gábor et al. (Aug. 2016). "Human pyramidal to interneuron synapses are mediated by multi-vesicular release and multiple docked vesicles". In:

eLife 5. ISSN: 2050-084X. DOI: 10.7554/elife.18167. URL: http://dx.doi.org/10.7554/eLife.18167.

Morelli, Emanuela et al. (Nov. 2014). “Environmental enrichment restores CA1 hippocampal LTP and reduces severity of seizures in epileptic mice”. In: *Experimental Neurology* 261, pp. 320–327. ISSN: 0014-4886. DOI: 10.1016/j.expneurol.2014.05.010. URL: http://dx.doi.org/10.1016/j.expneurol.2014.05.010.

Moreno-Velasquez, Laura et al. (May 2020). “Circuit-Specific Dendritic Development in the Piriform Cortex”. In: *eneuro* 7.3, ENEURO.0083–20.2020. ISSN: 2373-2822. DOI: 10.1523/eneuro.0083-20.2020. URL: http://dx.doi.org/10.1523/ENEURO.0083-20.2020.

Motley, Sarah E. et al. (Oct. 2018). “Selective Loss of Thin Spines in Area 7a of the Primate Intraparietal Sulcus Predicts Age-Related Working Memory Impairment”. In: *The Journal of Neuroscience* 38.49, pp. 10467–10478. ISSN: 1529-2401. DOI: 10.1523/jneurosci.1234-18.2018. URL: http://dx.doi.org/10.1523/JNEUROSCI.1234-18.2018.

Müller, Michaela Kerstin et al. (Oct. 2018). “NMDA receptors mediate synaptic depression, but not spine loss in the dentate gyrus of adult amyloid Beta (A) overexpressing mice”. In: *Acta Neuropathologica Communications* 6.1. ISSN: 2051-5960. DOI: 10.1186/s40478-018-0611-4. URL: http://dx.doi.org/10.1186/s40478-018-0611-4.

Murase, Sachiko et al. (June 2015). “Matrix Metalloproteinase-9 Regulates Neuronal Circuit Development and Excitability”. In: *Molecular Neurobiology* 53.5, pp. 3477–3493. ISSN: 1559-1182. DOI: 10.1007/s12035-015-9295-y. URL: http://dx.doi.org/10.1007/s12035-015-9295-y.

Nash, Amelia et al. (Nov. 2019). “Lack of Sez6 Family Proteins Impairs Motor Functions, Short-Term Memory, and Cognitive Flexibility and Alters Dendritic Spine Properties”. In: *Cerebral Cortex* 30.4, pp. 2167–2184. ISSN: 1460-2199. DOI: 10.1093/cercor/bhz230. URL: http://dx.doi.org/10.1093/cercor/bhz230.

Nassar, MÃ©rie et al. (May 2015). “Diversity and overlap of parvalbumin and somatostatin expressing interneurons in mouse presubiculum”. In: *Frontiers in Neural Circuits* 9. ISSN: 1662-5110. DOI: 10.3389/fncir.2015.00020. URL: http://dx.doi.org/10.3389/fncir.2015.00020.

Nedelescu, Hermina, Mohamed Abdelhack, and Arwel T. Pritchard (Jan. 2018). “Regional differences in Purkinje cell morphology in the cerebellar vermis of male mice”. In: *Journal of Neuroscience Research* 96.9, pp. 1476–1489. ISSN: 1097-4547. DOI: 10.1002/jnr.24206. URL: http://dx.doi.org/10.1002/jnr.24206.

Nelissen, Thom P. et al. (Feb. 2018). “CD38 is Required for Dendritic Organization in Visual Cortex and Hippocampus”. In: *Neuroscience* 372, pp. 114–125. ISSN: 0306-4522. DOI: 10.1016/j.neuroscience.2017.12.050. URL: http://dx.doi.org/10.1016/j.neuroscience.2017.12.050.

Nobili, Annalisa et al. (Apr. 2017). “Dopamine neuronal loss contributes to memory and reward dysfunction in a model of Alzheimer’s disease”. In:

Nature Communications 8.1. ISSN: 2041-1723. DOI: 10.1038/ncomms14727. URL: http://dx.doi.org/10.1038/ncomms14727.

Nörenberg, Anja et al. (Dec. 2009). “Distinct nonuniform cable properties optimize rapid and efficient activation of fast-spiking GABAergic interneurons”. In: *Proceedings of the National Academy of Sciences* 107.2, pp. 894–899. ISSN: 1091-6490. DOI: 10.1073/pnas.0910716107. URL: http://dx.doi.org/10.1073/pnas.0910716107.

Oaks, Adam W. et al. (Jan. 2016). “Cc2d1a Loss of Function Disrupts Functional and Morphological Development in Forebrain Neurons Leading to Cognitive and Social Deficits”. In: *Cerebral Cortex* 27.2, pp. 1670–1685. ISSN: 1460-2199. DOI: 10.1093/cercor/bhw009. URL: http://dx.doi.org/10.1093/cercor/bhw009.

Oboti, L. et al. (Feb. 2009). “Integration and sensory experience-dependent survival of newly-generated neurons in the accessory olfactory bulb of female mice”. In: *European Journal of Neuroscience* 29.4, pp. 679–692. ISSN: 1460-9568. DOI: 10.1111/j.1460-9568.2009.06614.x. URL: http://dx.doi.org/10.1111/j.1460-9568.2009.06614.x.

Ohgomori, Tomohiro et al. (Apr. 2016). “Comparative morphometric analysis of microglia in the spinal cord of¡scp¿SOD¡/scp¿1G93Atransgenic mouse model of amyotrophic lateral sclerosis”. In: *European Journal of Neuroscience* 43.10. Ed. by Yoland Smith, pp. 1340–1351. ISSN: 1460-9568. DOI: 10.1111/ejn.13227. URL: http://dx.doi.org/10.1111/ejn.13227.

Palmer, Lucy M et al. (Feb. 2014). “NMDA spikes enhance action potential generation during sensory input”. In: *Nature Neuroscience* 17.3, pp. 383–390. ISSN: 1546-1726. DOI: 10.1038/nn.3646. URL: http://dx.doi.org/10.1038/nn.3646.

Petit-Pedrol, Mar et al. (Oct. 2018). “LGI1 antibodies alter Kv1.1 and AMPA receptors changing synaptic excitability, plasticity and memory”. In: *Brain*. ISSN: 1460-2156. DOI: 10.1093/brain/awy253. URL: http://dx.doi.org/10.1093/brain/awy253.

Piirainen, Sami et al. (June 2021). “Microglia contribute to social behavioral adaptation to chronic stress”. In: *Glia* 69.10, pp. 2459–2473. ISSN: 1098-1136. DOI: 10.1002/glia.24053. URL: http://dx.doi.org/10.1002/glia.24053.

Platschek, Steffen et al. (Feb. 2016). “A general homeostatic principle following lesion induced dendritic remodeling”. In: *Acta Neuropathologica Communications* 4.1. ISSN: 2051-5960. DOI: 10.1186/s40478-016-0285-8. URL: http://dx.doi.org/10.1186/s40478-016-0285-8.

Prichard, Ashley et al. (Aug. 2023). “Brain rhythms control microglial response and cytokine expression via NF-B signaling”. In: *Science Advances* 9.32. ISSN: 2375-2548. DOI: 10.1126/sciadv.adf5672. URL: http://dx.doi.org/10.1126/sciadv.adf5672.

Putatunda, Raj et al. (Oct. 2018). “Adult neurogenic deficits in HIV-1 Tg26 transgenic mice”. In: *Journal of Neuroinflammation* 15.1. ISSN: 1742-2094. DOI: 10.1186/s12974-018-1322-2. URL: http://dx.doi.org/10.1186/s12974-018-1322-2.

Qin, Luye et al. (Feb. 2014). "An Adaptive Role for BDNF Val66Met Polymorphism in Motor Recovery in Chronic Stroke". In: *The Journal of Neuroscience* 34.7, pp. 2493–2502. ISSN: 1529-2401. DOI: 10.1523/jneurosci.4140-13.2014. URL: http://dx.doi.org/10.1523/JNEUROSCI.4140-13.2014.

Radman, Thomas et al. (Oct. 2009). "Role of cortical cell type and morphology in subthreshold and suprathreshold uniform electric field stimulation in vitro". In: *Brain Stimulation* 2.4, 215–228.e3. ISSN: 1935-861X. DOI: 10.1016/j.brs.2009.03.007. URL: http://dx.doi.org/10.1016/j.brs.2009.03.007.

Rivera, Carolina Caban et al. (Dec. 2023). "The ventral hippocampus and nucleus accumbens as neural substrates for cocaine contextual memory reconsolidation". In: DOI: 10.1101/2023.11.29.569314. URL: http://dx.doi.org/10.1101/2023.11.29.569314.

Rodrigues-Neves, Ana Catarina et al. (Feb. 2021). "Retina and Brain Display Early and Differential Molecular and Cellular Changes in the 3xTg-AD Mouse Model of Alzheimer's Disease". In: *Molecular Neurobiology* 58.7, pp. 3043–3060. ISSN: 1559-1182. DOI: 10.1007/s12035-021-02316-x. URL: http://dx.doi.org/10.1007/s12035-021-02316-x.

Rotaru, Diana C. et al. (Mar. 2015). "Functional properties of GABA synaptic inputs onto GABA neurons in monkey prefrontal cortex". In: *Journal of Neurophysiology* 113.6, pp. 1850–1861. ISSN: 1522-1598. DOI: 10.1152/jn.00799.2014. URL: http://dx.doi.org/10.1152/jn.00799.2014.

Routh, Brandy N. et al. (Oct. 2009). "Anatomical and Electrophysiological Comparison of CA1 Pyramidal Neurons of the Rat and Mouse". In: *Journal of Neurophysiology* 102.4, pp. 2288–2302. ISSN: 1522-1598. DOI: 10.1152/jn.00082.2009. URL: http://dx.doi.org/10.1152/jn.00082.2009.

Rovira-Esteban, Laura et al. (Apr. 2017). "Morphological and physiological properties of CCK/CB1R-expressing interneurons in the basal amygdala". In: *Brain Structure and Function* 222.8, pp. 3543–3565. ISSN: 1863-2661. DOI: 10.1007/s00429-017-1417-z. URL: http://dx.doi.org/10.1007/s00429-017-1417-z.

Rumbell, Timothy H. et al. (Apr. 2016). "Automated evolutionary optimization of ion channel conductances and kinetics in models of young and aged rhesus monkey pyramidal neurons". In: *Journal of Computational Neuroscience* 41.1, pp. 65–90. ISSN: 1573-6873. DOI: 10.1007/s10827-016-0605-9. URL: http://dx.doi.org/10.1007/s10827-016-0605-9.

Santos, Victor R. et al. (Dec. 2017). "PTEN deletion increases hippocampal granule cell excitability in male and female mice". In: *Neurobiology of Disease* 108, pp. 339–351. ISSN: 0969-9961. DOI: 10.1016/j.nbd.2017.08.014. URL: http://dx.doi.org/10.1016/j.nbd.2017.08.014.

Sanz-Morello, Berta et al. (Aug. 2020). "Complex ¡scp¿IV¡/scp¿ subunit isoform ¡scp¿COX¡/scp¿ 6A2 protects fast-spiking interneurons from oxidative stress and supports their function". In: *The EMBO Journal* 39.18. ISSN: 1460-2075. DOI: 10.15252/embj.2020105759. URL: http://dx.doi.org/10.15252/embj.2020105759.

Schoenfeld, Timothy J. et al. (Oct. 2023). “Rewarded Maze Training Increases Approach Behavior in Rats Through Neurogenesis-Dependent Growth of Ventral Hippocampus–Prelimbic Circuits”. In: *Biological Psychiatry Global Open Science* 3.4, pp. 725–733. ISSN: 2667-1743. DOI: 10.1016/j.bpsgos.2023.04.003. URL: http://dx.doi.org/10.1016/j.bpsgos.2023.04.003.

Schulz, Jan M. et al. (Aug. 2021). “GABAB Receptor-Mediated Regulation of Dendro-Somatic Synergy in Layer 5 Pyramidal Neurons”. In: *Frontiers in Cellular Neuroscience* 15. ISSN: 1662-5102. DOI: 10.3389/fncel.2021.718413. URL: http://dx.doi.org/10.3389/fncel.2021.718413.

Sepulveda-Rodriguez, Alberto et al. (Mar. 2019). “Electroconvulsive Shock Enhances Responsive Motility and Purinergic Currents in Microglia in the Mouse Hippocampus”. In: *eneuro* 6.2, ENEURO.0056–19.2019. ISSN: 2373-2822. DOI: 10.1523/eneuro.0056-19.2019. URL: http://dx.doi.org/10.1523/ENEURO.0056-19.2019.

Shi, Yuying et al. (July 2023). “Laminar and dorsoventral organization of layer 1 interneuronal microcircuitry in superficial layers of the medial entorhinal cortex”. In: *Cell Reports* 42.7, p. 112782. ISSN: 2211-1247. DOI: 10.1016/j.celrep.2023.112782. URL: http://dx.doi.org/10.1016/j.celrep.2023.112782.

Soloway, A.S. et al. (Feb. 2002). “Dendritic morphology of callosal and ipsilateral projection neurons in monkey prefrontal cortex”. In: *Neuroscience* 109.3, pp. 461–471. ISSN: 0306-4522. DOI: 10.1016/s0306-4522(01)00507-3. URL: http://dx.doi.org/10.1016/S0306-4522(01)00507-3.

Soto-Diaz, Katiria et al. (Nov. 2021). “Treatment With the CSF1R Antagonist GW2580, Sensitizes Microglia to Reactive Oxygen Species”. In: *Frontiers in Immunology* 12. ISSN: 1664-3224. DOI: 10.3389/fimmu.2021.734349. URL: http://dx.doi.org/10.3389/fimmu.2021.734349.

Sousa, Aline A. de et al. (June 2015). “Three-dimensional morphometric analysis of microglial changes in a mouse model of virus encephalitis: age and environmental influences”. In: *European Journal of Neuroscience* 42.4, pp. 2036–2050. ISSN: 1460-9568. DOI: 10.1111/ejn.12951. URL: http://dx.doi.org/10.1111/ejn.12951.

Stedehouder, Jeffrey et al. (Nov. 2019). “Local axonal morphology guides the topography of interneuron myelination in mouse and human neocortex”. In: *eLife* 8. ISSN: 2050-084X. DOI: 10.7554/elife.48615. URL: http://dx.doi.org/10.7554/eLife.48615.

Stokes, Caleb C. A., Corinne M. Teeter, and Jeffry S. Isaacson (Nov. 2014). “Single dendrite-targeting interneurons generate branch-specific inhibition”. In: *Frontiers in Neural Circuits* 8. ISSN: 1662-5110. DOI: 10.3389/fncir.2014.00139. URL: http://dx.doi.org/10.3389/fncir.2014.00139.

Sun, Qian-Quan (Nov. 2009). “Experience-Dependent Intrinsic Plasticity in Interneurons of Barrel Cortex Layer IV”. In: *Journal of Neurophysiology* 102.5, pp. 2955–2973. ISSN: 1522-1598. DOI: 10.1152/jn.00562.2009. URL: http://dx.doi.org/10.1152/jn.00562.2009.

Szegedi, Viktor et al. (Nov. 2016). “Plasticity in Single Axon Glutamatergic Connection to GABAergic Interneurons Regulates Complex Events in

the Human Neocortex". In: *PLOS Biology* 14.11. Ed. by Alberto Bacci, e2000237. ISSN: 1545-7885. DOI: 10.1371/journal.pbio.2000237. URL: http://dx.doi.org/10.1371/journal.pbio.2000237.

Szoboszlay, Miklos et al. (June 2016). "Functional Properties of Dendritic Gap Junctions in Cerebellar Golgi Cells". In: *Neuron* 90.5, pp. 1043–1056. ISSN: 0896-6273. DOI: 10.1016/j.neuron.2016.03.029. URL: http://dx.doi.org/10.1016/j.neuron.2016.03.029.

Tan, Shawn et al. (Oct. 2018). "Postnatal TrkB ablation in corticolimbic interneurons induces social dominance in male mice". In: *Proceedings of the National Academy of Sciences* 115.42. ISSN: 1091-6490. DOI: 10.1073/pnas.1812083115. URL: http://dx.doi.org/10.1073/pnas.1812083115.

Tensaouti, Yacine et al. (July 2018). "ApoE Regulates the Development of Adult Newborn Hippocampal Neurons". In: *eneuro* 5.4, ENEURO.0155–18.2018. ISSN: 2373-2822. DOI: 10.1523/eneuro.0155-18.2018. URL: http://dx.doi.org/10.1523/eneuro.0155-18.2018.

Testa-Silva, Guilherme et al. (Nov. 2014). "High Bandwidth Synaptic Communication and Frequency Tracking in Human Neocortex". In: *PLoS Biology* 12.11. Ed. by Idan Segev, e1002007. ISSN: 1545-7885. DOI: 10.1371/journal.pbio.1002007. URL: http://dx.doi.org/10.1371/journal.pbio.1002007.

Tikhonova, Tatiana B. et al. (Sept. 2018). "Cell Type- and Layer-Specific Muscarinic Potentiation of Excitatory Synaptic Drive onto Parvalbumin Neurons in Mouse Prefrontal Cortex". In: *eneuro* 5.5, ENEURO.0208–18.2018. ISSN: 2373-2822. DOI: 10.1523/eneuro.0208-18.2018. URL: http://dx.doi.org/10.1523/ENEURO.0208-18.2018.

Tischfield, David J. et al. (July 2017). "Loss of the neurodevelopmental gene Zswim6 alters striatal morphology and motor regulation". In: *Neurobiology of Disease* 103, pp. 174–183. ISSN: 0969-9961. DOI: 10.1016/j.nbd.2017.04.013. URL: http://dx.doi.org/10.1016/j.nbd.2017.04.013.

Toth, Eszter et al. (May 2021). "A morphological analysis of activity-dependent myelination and myelin injury in transitional oligodendrocytes". In: *Scientific Reports* 11.1. ISSN: 2045-2322. DOI: 10.1038/s41598-021-88887-0. URL: http://dx.doi.org/10.1038/s41598-021-88887-0.

Tripathy, Shreejoy J. et al. (June 2015). "Brain-wide analysis of electrophysiological diversity yields novel categorization of mammalian neuron types". In: *Journal of Neurophysiology* 113.10, pp. 3474–3489. ISSN: 1522-1598. DOI: 10.1152/jn.00237.2015. URL: http://dx.doi.org/10.1152/jn.00237.2015.

Tyan, Leonid et al. (Mar. 2014). "Dendritic Inhibition Provided by Interneuron-Specific Cells Controls the Firing Rate and Timing of the Hippocampal Feedback Inhibitory Circuitry". In: *The Journal of Neuroscience* 34.13, pp. 4534–4547. ISSN: 1529-2401. DOI: 10.1523/jneurosci.3813-13.2014. URL: http://dx.doi.org/10.1523/JNEUROSCI.3813-13.2014.

Urrego, Diana, Julieta Troncoso, and Alejandro Múnera (2015). "Layer 5 Pyramidal Neurons' Dendritic Remodeling and Increased Microglial Density in Primary Motor Cortex in a Murine Model of Facial Paralysis". In: *BioMed*

Research International 2015, pp. 1–11. ISSN: 2314-6141. DOI: 10.1155/2015/482023. URL: http://dx.doi.org/10.1155/2015/482023.

Valero, Manuel et al. (July 2015). "Determinants of different deep and superficial CA1 pyramidal cell dynamics during sharp-wave ripples". In: *Nature Neuroscience* 18.9, pp. 1281–1290. ISSN: 1546-1726. DOI: 10.1038/nn.4074. URL: http://dx.doi.org/10.1038/nn.4074.

Varga, Csaba, Mikko Oijala, et al. (Nov. 2014). "Functional fission of parvalbumin interneuron classes during fast network events". In: *eLife* 3. ISSN: 2050-084X. DOI: 10.7554/elife.04006. URL: http://dx.doi.org/10.7554/eLife.04006.

Varga, Csaba, Gabor Tamas, et al. (Mar. 2015). "Molecular and Electrophysiological Characterization of GABAergic Interneurons Expressing the Transcription Factor COUP-TFII in the Adult Human Temporal Cortex". In: *Cerebral Cortex* 25.11, pp. 4430–4449. ISSN: 1460-2199. DOI: 10.1093/cercor/bhv045. URL: http://dx.doi.org/10.1093/cercor/bhv045.

Vlasits, Anna L. et al. (Mar. 2016). "A Role for Synaptic Input Distribution in a Dendritic Computation of Motion Direction in the Retina". In: *Neuron* 89.6, pp. 1317–1330. ISSN: 0896-6273. DOI: 10.1016/j.neuron.2016.02.020. URL: http://dx.doi.org/10.1016/j.neuron.2016.02.020.

Walker, F. et al. (Nov. 2016). "Parvalbumin- and vasoactive intestinal polypeptide-expressing neocortical interneurons impose differential inhibition on Martinotti cells". In: *Nature Communications* 7.1. ISSN: 2041-1723. DOI: 10.1038/ncomms13664. URL: http://dx.doi.org/10.1038/ncomms13664.

Wang, Chi et al. (Nov. 2020). "Tactile modulation of memory and anxiety requires dentate granule cells along the dorsoventral axis". In: *Nature Communications* 11.1. ISSN: 2041-1723. DOI: 10.1038/s41467-020-19874-8. URL: http://dx.doi.org/10.1038/s41467-020-19874-8.

Wang, Han-Ying et al. (Dec. 2015). "RBFOX3/NeuN is Required for Hippocampal Circuit Balance and Function". In: *Scientific Reports* 5.1. ISSN: 2045-2322. DOI: 10.1038/srep17383. URL: http://dx.doi.org/10.1038/srep17383.

Wang, Xiaojun et al. (Mar. 2019). "Genetic Single Neuron Anatomy Reveals Fine Granularity of Cortical Axo-Axonic Cells". In: *Cell Reports* 26.11, 3145–3159.e5. ISSN: 2211-1247. DOI: 10.1016/j.celrep.2019.02.040. URL: http://dx.doi.org/10.1016/j.celrep.2019.02.040.

Watson, K.K., T.K. Jones, and J.M. Allman (Jan. 2006). "Dendritic architecture of the von Economo neurons". In: *Neuroscience* 141.3, pp. 1107–1112. ISSN: 0306-4522. DOI: 10.1016/j.neuroscience.2006.04.084. URL: http://dx.doi.org/10.1016/j.neuroscience.2006.04.084.

Yamada, Jun et al. (Dec. 2016). "Cell type- and region-specific enhancement of adult hippocampal neurogenesis by daidzein in middle-aged female mice". In: *Neuropharmacology* 111, pp. 92–106. ISSN: 0028-3908. DOI: 10.1016/j.neuropharm.2016.08.036. URL: http://dx.doi.org/10.1016/j.neuropharm.2016.08.036.

Yang, Cheng-Yi et al. (Apr. 2019). "Conditional Deletion of CC2D1A Reduces Hippocampal Synaptic Plasticity and Impairs Cognitive Function through

Rac1 Hyperactivation". In: *The Journal of Neuroscience* 39.25, pp. 4959–4975. ISSN: 1529-2401. DOI: 10.1523/jneurosci.2395-18.2019. URL: http://dx.doi.org/10.1523/JNEUROSCI.2395-18.2019.

Zaitsev, A. V. et al. (July 2012). "Electrophysiological classes of layer 2/3 pyramidal cells in monkey prefrontal cortex". In: *Journal of Neurophysiology* 108.2, pp. 595–609. ISSN: 1522-1598. DOI: 10.1152/jn.00859.2011. URL: http://dx.doi.org/10.1152/jn.00859.2011.

Zhang, Xiaomin, Alois Schlögl, and Peter Jonas (Sept. 2020). "Selective Routing of Spatial Information Flow from Input to Output in Hippocampal Granule Cells". In: *Neuron* 107.6, 1212–1225.e7. ISSN: 0896-6273. DOI: 10.1016/j.neuron.2020.07.006. URL: http://dx.doi.org/10.1016/j.neuron.2020.07.006.
